# *Trichoderma asperellum* T42 Reprograms Tobacco for Enhanced Nitrogen Utilization Efficiency and Plant Growth When Fed with N Nutrients

**DOI:** 10.3389/fpls.2018.00163

**Published:** 2018-02-20

**Authors:** Bansh N. Singh, Padmanabh Dwivedi, Birinchi K. Sarma, Gopal S. Singh, Harikesh B. Singh

**Affiliations:** ^1^Institute of Environment and Sustainable Development, Banaras Hindu University, Varanasi, India; ^2^Department of Plant Physiology, Institute of Agricultural Sciences, Banaras Hindu University, Varanasi, India; ^3^Department of Mycology and Plant Pathology, Institute of Agricultural Sciences, Banaras Hindu University, Varanasi, India

**Keywords:** ammonium, nitrate nutrition, nitrate transporter genes, nitric oxide, *Trichoderma asperellum* T42, root architecture

## Abstract

*Trichoderma* spp., are saprophytic fungi that can improve plant growth through increased nutrient acquisition and change in the root architecture. In the present study, we demonstrate that *Trichoderma asperellum* T42 mediate enhancement in host biomass, total nitrogen content, nitric oxide (NO) production and cytosolic Ca^2+^ accumulation in tobacco. T42 inoculation enhanced lateral root, root hair length, root hair density and root/shoot dry mass in tobacco under deprived nutrients condition. Interestingly, these growth attributes were further elevated in presence of T42 and supplementation of NO_3_^-^ and NH_4_^+^ nutrients to tobacco at 40 and 70 days, particularly in NO_3_^-^ supplementation, whereas no significant increment was observed in *nia30* mutant. In addition, NO production was more in tobacco roots in T42 inoculated plants fed with NO_3_^-^ nutrient confirming NO generation was dependent on NR pathway. NO_3_^-^ dependent NO production contributed to increase in lateral root initiation, Ca^2+^ accumulation and activities of nitrate transporters (NRTs) in tobacco. Higher activities of several NRT genes in response to T42 and N nutrients and suppression of ammonium transporter (*AMT1*) suggested that induction of high affinity NRTs help NO_3_^-^ acquisition through roots of tobacco. Among the NRTs *NRT2*.*1* and *NRT2.2* were more up-regulated compared to the other NRTs. Addition of sodium nitroprusside (SNP), relative to those supplied with NO_3_^-^/NH_4_^+^ nutrition and T42 treated plants singly, and with application of NO inhibitor, cPTIO, confirmed the altered NO fluorescence intensity in tobacco roots. Our findings suggest that T42 promoted plant growth significantly ant N content in the tobacco plants grown under N nutrients, notably higher in NO_3_^-^, providing insight of the strategy for not only tobacco but probably for other crops as well to adapt to fluctuating nitrate availability in soil.

## Introduction

Nitrate is one of the most important sources of nitrogen, not only for plant development but also for plant–microbe interactions (Dordas, 2008). Several reports have demonstrated that nitrate not only takes part in plant growth and development but its presence affects several other physiological processes such as disease management (Gupta et al., 2013), root organogenesis (Sun et al., 2015; Ruffel et al., 2016), flowering induction (Marín et al., 2011), crop growth and yield increment (Krapp et al., 2014). In agricultural systems, farmers generally prefer NO_3_^-^, NH_4_^+^ or a combination of both as N fertilizers for plant growth. Subsequently, NR activity reduces NO_3_^-^ to NO_2_^-^ and finally converted into NH_4_^+^, which is involved in amino acid biosynthesis through glutamine synthetase. Parallely, NH_4_^+^ can also incorporate in the soil biosystem either by degrading organic matter by the microorganisms or fixing atmospheric N. First time, Müller (1983) noticed that *nia1/nia2* (*nia28*) double mutant tobacco plants were unable to utilize nitrate as a N source from medium whereas *nia30* seedlings utilized nitrate at a very small rate. Recently, several researches had highlighted that nitrate participate in the regulation of NRT genes which help in uptake, transport and assimilation of nitrate (Medici and Krouk, 2014; Pii et al., 2014, 2016a; Noguero and Lacombe, 2016). Since, concentrations of nitrate vary from μM to mM in soil, therefore, for optimum uptake of nitrate from soil, plants have modulating nitrogen acquisition capacity and altering their own system such as root architecture (Miller et al., 2007; Song et al., 2013; Manoli et al., 2014). Several nitrate and AMTs are known which maximized uptake efficiency of nitrate through root system (Negi et al., 2008; Krapp, 2015). Based on their N uptake efficiency, NRTs are categorized into two groups. The low-affinity transporter allows the high capacity of external nitrate uptake (>0.5 mM) while high-affinity transporters (HATS) are essential for the uptake when the external concentration of NO_3_^-^ is low (<0.5 mM) (Miller et al., 2007). Several NRTs have been identified in plant roots with a high-affinity for nitrate (Krouk et al., 2010; Kiba et al., 2012; Manoli et al., 2014; Pii et al., 2014, 2016a; Sun et al., 2015). However, our understanding on plants sensing of fluctuating soil nitrate concentrations and the signal induction that affects the root architecture remains narrow.

Nitric oxide (NO) is a signaling molecule generated by NR activity which regulates root traits development (Pii et al., 2007; Zhao et al., 2007; Manoli et al., 2014; Trevisan et al., 2014). Several evidences suggested that induction of NO signal is not only limited to root development but also regulated many plant physiological processes during nutrient assimilation (Correa-Aragunde et al., 2004; Chen et al., 2010; Chen and Kao, 2012; Meng et al., 2012; Nacry et al., 2013). Stöhr and Ullrich (2002) reported that root apex is the first site where NO is produced by NR activity during N acquisition. Interactions of NO with plant hormone like auxin further facilitated LR formation in tomato (Correa-Aragunde et al., 2004). But feedback inhibition of NR by NO was also noted in leaves of wheat (Rosales et al., 2011). Indeed, NR activation or inhibition by NO depends on external nitrate concentration in root of tomato (Jin et al., 2009). Furthermore, several reports highlighted that NO not only crosstalks with plant hormones in root organogenesis but also induced [Ca^2+^]cyt second messenger in the cGMP-dependent signaling pathways in plants that helps in cell wall synthesis, cellular homeostasis (Lamattina et al., 2003). White (2001) suggested that NO elevated release of Ca^2+^ in cytosol during root cell and root hair elongation. Similar case was also reported during root formation mediated through NO that induced Ca^2+^ signaling (Pagnussat et al., 2003). But complete knowledge about NO regulation in N uptake and interaction with Ca^2+^ in response to varying external nitrate concentrations is limited.

The agricultural soil has lots of beneficial microorganisms (*Pseudomonas, Bacillus*, Arbuscular mycorrhiza, *Trichoderma* sp., etc.) which are sustainably used for plant growth and development (Shoresh et al., 2010; Jain et al., 2012; Singh et al., 2015). They affect plant by increasing nutrient efficacy, nutrient recycling, seed germination, establishing induced systemic resistance and releasing plant growth promoting agents (Jeffries et al., 2003; Shoresh et al., 2010; Azarmi et al., 2011; Singh et al., 2014; Pii et al., 2015, 2016b; Giles et al., 2016; Scagliola et al., 2016). Plant growth promoting fungal interaction with plant roots does not have a similar efficiency for nutrient uptake throughout the life cycle of plant. Probably, microbial efficiency for nutrient uptake after reproductive phase of plants gets reduced (Jensen, 1986). In addition, *Trichoderma* sp. is one of the important rhizosphere microorganisms that can colonize at the outer epidermal layers of the roots and solubilize various nutrients such as N, Fe, Cu, Zn, Mn in soils (Singh et al., 2014). Similarly, *Trichoderma harzianum* T22 is reported to increase nitrogen utilization efficiency in maize (Harman, 2000). Plant nitrogen utilization increases with increased growth and yield at a certain point, but the response is not associated with increased yields in rising nitrogen fertilization. Seed treatment of wheat with *Trichoderma* has also increased yields (Harman, 2006). Besides wide range of effects on plant growth and yields, *Trichoderma* formulations have been preferred over chemical fertilizers in agriculture sector (Viterbo and Horwitz, 2010). Several evidences have shown that microbes like *Trichoderma* spp. are directly involved in NO generation mediated through *NR* expression in plants (Sherameti et al., 2005). Gupta et al. (2014) reported that *T. asperelloides* exhibited NO emission during interaction with plant roots. The ability of *Trichoderma* to elevate NO production through NR is well known, although specific knowledge of regulation of NRTs/AMTs and the induction of signal transduction during N acquisition and assimilation in the presence of N fertilizers remains limited. Given the mechanisms of action of *Trichoderma* strains and their broad efficiencies to enhance nitrogen utilization capacity in plants, a new strain was selected for improvement of nitrogen utilization efficiency in tobacco. We investigated the possible roles of *T. asperellum* T42 during interaction with tobacco roots and analyzed nitrogen acquisition and assimilation capacity in tobacco roots and correlated the effects with plant growth through possible mediation of NO under supplementation of different forms of N nutrients.

## Materials and Methods

### Microorganism, Tobacco Seeds and Experimental Setup

Seeds of tobacco (*Nicotiana tabacum* cv. *Xanthi*) were procured from CTRI, Rajahmundry, A.P., India. Seeds of a nitrate reductase (NR)-deficient *nia30* mutant (cv. ‘Gatersleben’) were obtained from Prof. W. M. Kaiser, University of Würzburg, Germany. *T. asperellum* T42 (Gene bank accession: JN128894), referred herewith as T42 strain, was used as wild-type strain throughout this study. The T42 strain was used as biofertilizer for seed bio-priming of host tobacco seeds and propagated on potato dextrose agar (PDA, Himedia) medium at 28 ± 2°C.

Tobacco seeds were disinfected with 0.15% sodium hypochlorite and 0.01% of Tween-20 for 2 min. The seeds were bio-primed with T42 according to Yadav et al. (2013). Briefly, an aqueous suspension of T42 strain containing 1 × 10^6^ cfu mL^-1^ was prepared in distilled water containing 1% CMC (carboxymethyl cellulose, Himedia). The seeds were dipped in the spore suspension for an hour and then air-dried under a laminar flow hood for 7–8 h. T42 strain treated tobacco seeds were allowed to germinate on petriplates containing moist filter paper soaked with and without N (nitrate and ammonium) nutrient solution under continuous day/night regime of 16/8 h, 22°C/20°C, and a relative humidity of 70% with artificial light of PPDF of 350–400 μmol m^-2^s^-1^. Non-bio-primed tobacco plants grown under nutrient deprived conditions served as a control. After 2 weeks, plants were transferred in Perspex tubes containing 1.8 L hydroponic solution (Planchet et al., 2005) for next 70 days. Hydroponic culture solution with nitrate (pH 6.3) supplement consisted of 10 mM KNO_3_, 1 mM CaCl_2_, 1 mM MgSO_4_, 25 μM NaFe-EDTA, 0.5 mM K_2_HPO_4_, 1 mM KH_2_PO_4_, while for ammonium nutrition, 3 mM NH_4_Cl, 1 mM CaCl_2_, 1 mM MgSO_4_, 25 μM NaFe-EDTA, 0.5 mM K_2_HPO_4_, 1 mM KH_2_PO_4_. Trace elements were used as described earlier (Johnson et al., 1957). The NR-deficient *nia30* mutant plants were grown in nutrient solution having 10 mM KNO_3_, 3 mM NH_4_Cl, 1 mM CaCl_2_, 2 mM MgSO_4_, 25 μM Fe-EDTA, 2 mM KH_2_PO_4_/K_2_HPO_4_ and trace elements. The nutrient solutions were changed every 2 days during the course of experiment. Further, an experiment was conducted to determine the efficiency of *NRT2.1, NR* gene and NO emission in roots. A separate set of control tobacco plants were exposed to SNP (sodium nitroprusside, Sigma–Aldrich). The chemical composition of 10–200 μM SNP as NO donor was added in addition to NH_4_^+^ fed plants for 48 h. Oxidation of NH_4_^+^ was prevented by adding 10 μM dicyanimide in each pot. However, addition of 200 μM cPTIO [2-(4-carboxyphenyl)-4,4,5,5-tetramethylimidazoline-1-oxyl-3-oxide; Sigma–Aldrich] was used as a NO scavenger. Three independent biological replicates were kept in each experiment. The tobacco plants were grown in nutrient solution filled in Perspex tubes (0.5 m high, 15 cm inner diameter) covered with a black plastic sheet to prevent light in a plant growth chamber. The tubes were filled with 1.8 L nitrogen nutrient solution as described above for each treated tobacco plant.

Three week-old tobacco plantlets were grown hydroponically with different concentrations of 10–200 mM NO_3_^-^ and 3–200 mM NH_4_^+^ nutrients as N source with other macronutrients and trace elements as discussed below for next 30 days. Nutrient media were replaced twice in a week. After 30 days, root proliferation in all concentrations was observed (Supplementary Figure S1).

### Sampling, Measurements and Analyses

Length and diameter of tobacco roots were measured using traditional scale method at 40 and 70 days after bio-priming. Root intensity was calculated according to Thorup-Kristensen et al. (2009) and expressed as root intersection m^-1^ grid line. Root hairs from each replicate was randomly selected and carefully placed on petriplates. Root hair images were captured for all the tobacco roots on the main axis by ScopePhot (86×) camera fitted to an OLYMPUS OIC (105144) microscope at 10× magnification interfaced with a computer image board. Root hairs length was analyzed using ImageJ software. Root hair intensity and root hair length (RHL) were measured for more than 30 randomly selected root hairs. Root hair intensity was determined as the number of root hairs per mm root length. The root and shoot dry mass (RDM and SDM, respectively) were determined after oven drying at 65°C for 48 h.

### Total Nitrogen Content Determination

Total N content was determined in roots of tobacco plants from different treatments. Roots were oven dried at 60°C for 48 h and their dry weight was determined. N concentration from 100 mg dried/powdered root samples was estimated by semi-automatic nitrogen analyzer (Kjeldahl, Pelican, Kelplus-classic DX VATS, E), adapting Kjeldahl methods (Mishra et al., 2016).

### FT-IR Analysis of Root

FT-IR spectrometer (Bruker EQUINOX 55, Bruker, Ettlingen, Germany) with a range of infrared light source (4000–400 cm^-1^ wave numbers) was used to analyze the chemical finger-print of tobacco roots. The root samples were ground to a fine powder under high pressure and placed on a KBr microscope window and then examined. A background spectrum of the clear KBr window was recorded before acquisition of sample spectra and subtracted from the sample spectrum. To find more information about the sample’s homogeneity of the same treatment, spectra obtained from the same root samples (*n* = 3) were averaged and the average spectra further analyzed by the Origin8 software.

### NO Content in Tobacco Roots

Nitric oxide content in the root was detected using 10 μM DAF-FM DA (fluorophore 4, 5-diaminofluorescein-FM diacetate, Alexis Biochemicals, Gruenberg, Germany) as described elsewhere (Fernández-Marcos et al., 2011). Tobacco root were dipped in 10 μM DAF-FM dye (whose stock solution was prepared in 10 mM HEPES-KOH, pH 7.5), for 30 min at 25°C in a dark room. After incubation, roots were rinsed three times for 5 min with HEPES-KOH buffer. In order to verify participation of a NO donor (100 μM SNP) and/or a scavenger (200 μM cPTIO) on NO accumulation, SNP and cPTIO were applied to 70 days old tobacco plants grown in nitrate and ammonium nutrient as well as T42 treated plant including control. Root fluorescence image and intensity was analyzed by Nikon Eclipse 90i fluorescence microscope (Nikon Instruments Inc., United States) with 500 nm excitation and 515 nm emission. Fluorescence intensity was expressed as arbitrary fluorescence units (A.U.). Three roots were used for each condition and independent analysis for each treatment.

### Ca Accumulation in Tobacco Root

Roots from different treatments were collected and briefly washed with distilled water. The samples were then incubated in 10 μM Fluo-4 AM [4-(6-Acetoxymethoxy-2,7-difluoro-3-oxo-9-xanthenyl)-4′-methyl-2,2′-(ethylenedioxy)dianiline-N,N,N′, N′-tetraacetic acid tetra -kis (acetoxymethyl) ester; Sigma–Aldrich] in loading buffer [diluted from 5 mM stock solution in 10 mM MES-KOH; 2-(N-Morpholino) ethanesulfonic acid sodium salt, 4-Morpholineethanesulfonic acid sodium salt; pH 6.15] for 2 h at 4°C in darkness. Root samples were rinsed thrice with loading buffer to remove excess dye and kept at 24°C in the growth chamber for 1–2 h. Fluorescence intensity was increased when intracellular esterase removed AM from Fluo-4AM and bind to cytosolic Ca^2+^, measured at excitation (488 nm) and emission (510–545 nm) wavelength. All images were taken using Nikon Eclipse 90i fluorescence microscope (Nikon UK Ltd., Telford, United Kingdom). Higher concentration of Fluo-4AM may be due to binding of dye with other divalent cations like Mn^2+^, Pb^2+^, and Zn^2+^. For Ca^2+^ inhibitor studies, control plants were incubated in 10 mM Ca^2+^ channel blocker LaCl_3_ for 1 h prior to fluorescence imaging (Pei et al., 2000).

### RNA Extraction, Reverse Transcription, and Quantitative PCR of Roots

Root samples (500 mg) were collected at 70 days and pooled from three plants in each treatment. Total RNA was isolated from root as described by Patel et al. (2016). Two micrograms of RNA was digested with DNase I (Fermentas) and total RNA was used as template for first-strand cDNA synthesis with High Capacity cDNA Reverse Transcription Kit (Applied Biosystems, Monza, Italy). Experiments were conducted using SYBR Green chemistry (PowerUp^TM^ SYBR Green Master Mix, Applied Biosystems) with Applied Biosystems 7500 version 2.0.4. For each reaction, 50 ng cDNA was used as template. Reactions were performed with three biological replicates under the following conditions: an initial denaturation step (10 min at 95°C) followed by 40 cycles of denaturation at 95°C for 2 min, 40 repeats at 95°C for 20 s, 60°C for 30 s, and 72°C for 30 s. The data normalization with mean *C*_t_ (Cycle threshold) values of target and reference gene was calculated 2^-ΔΔ^*^C^*^T^ using established method (Schmittgen and Livak, 2008). For each transcript, the ratio between the expressions measured for a given treatment with own a control gene was considered.

Relative expression analysis was performed using six nitrogen acquisition and one NO response genes in tobacco. Primer sequences were designed using online software Primer3 (web tool version 0.4.0^1^). The whole sequences for corresponding genes were retrieved from NCBI. The primers and gene locus numbers for the *NtNRT1.2s* (AB102807), *NtNRT1.2t* (AB102808), *NtNRT1.1s* (AB102805), *NtNRT2.1* (AJ557583), *NtNRT2.2* (AJ557584), *NtAMT1* (KJ874416), *N. tabaccum nia-*1 for NR, *NtNIA1* (X14058.1), for internal control *N. tabacum* β-tubulin (KP316400.1) are listed in Supplementary Table S2. Transcript levels were normalized to expression of the tobacco β-tubulin, a housekeeping gene and results are expressed as fold increase or down-regulated as compared to control.

### Statistical Analysis

Values from different experiments shown in figures are mean ± standard error (SE) (*n* = 3). Statistical analyses were subjected to one way analysis of variance (ANOVA). For comparison between treatments, Duncan’s multiple range test at *P* < 0.05; *P* < 0.01 significance levels were applied and analyzed by SPSS ver. 16 (SPSS Inc., Chicago, IL, United States).

## Results and Discussion

### T42 and Nitrate Nutrition Promoted Root Architectures, Root/Shoot Dry Matter, and Thickness Higher in Wild-Type but to Lesser Extent in Tobacco *nia30* Mutant

**FIGURE 1:**
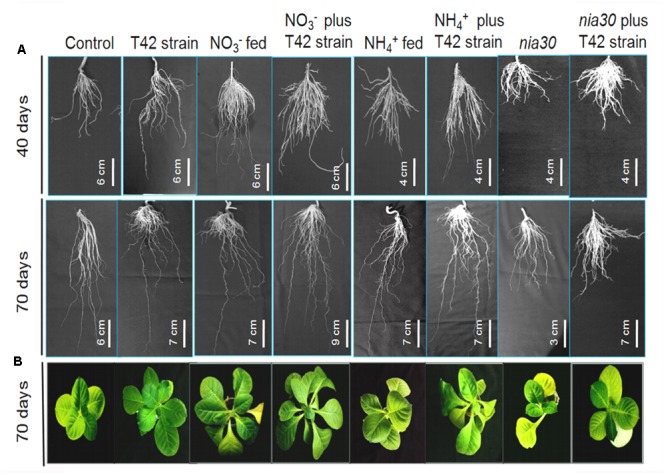
Root morphology variation in *Nicotiana tabacum* and nitrate reductase-(NR) *nia30* mutant at 40 and 70 days of growth **(A)** and plant morphology **(B)** at 70 days. Bars in different figures show measurement of root length.

Nitrogen is one of the major elements among macronutrients for plant life. Crops are strongly dependent on chemical fertilizer throughout the world, thus causing adverse effects on environmental quality. NH_4_NO_3_ and CO(NH_2_)_2_ are common fertilizers that have been extensively used in agriculture fields by farmers. Nitrate form is easily available to the plant, but due to the negative charge on clay particle, it is easily leached out of the soil to become a major source of eutrophication (Peng and Zhu, 2006). Nitrogen acquisition is a major factor for development and productivity of crop plants because it is present either in a complex form or very limited amount that cannot be easily available to plants. Therefore, scientists continue to develop such type of crops with improved nutrient uptake efficiency under low nitrogen input agriculture system (Robertson and Vitousek, 2009; Xu et al., 2012) as well as identifying beneficial microbes which show mutual symbiotic relationships with a broad range of commercial crops, thus ensuring better mineral nutrient acquisition efficiency by the plant. Root architecture generally influences nutrient acquisition through increased root distribution over the soil layers with increased surface area for nutrient uptake (Lynch, 2013; White et al., 2013). Especially, root hairs were more important component of root traits that facilitate mineral nutrient uptake in several plant species, particularly in plants experiencing low mineral nutrient status (Yan et al., 2004; Brown et al., 2012; Wang et al., 2016; Aceves-García et al., 2016). However, relative significance of root hairs to N acquisition in NO_3_^-^ and NH_4_^+^ fertilization in presence of beneficial rhizospheric fungi such as *Trichoderma* is still unclear. In the current experiment, inoculation of tobacco roots with *T. asperellum* T42 increased root differentiation comparatively more than control. Approximately, 109% increase in RDM was found in T42 bio-primed tobacco plants at 70 days after treatment compared to control, and no significant difference was observed in *nia30* mutant (**Table 1**). Similar to RDM, approximately 115% increase in SDM was noticed in T42 treatment. Quantification of the RDM and SDM indicated that T42 had more impact on tobacco plants and increased primary root length (PRL), RD and number of LR, particularly in NO_3_^-^ nutrient instead of NH_4_^+^ (**Figure 1**). Approximately 115% PRL, 168% RDM, and 149% SDM were increased in T42 inoculated tobacco roots fed with NO_3_^-^ nutrient at 70 days of growth (**Table 1**). Subsequently, PRL, RDM, and SDM recorded 52, 84, and 94% increment, respectively, in non-bio-primed T42 tobacco plants fed with NO_3_^-^ nutrient compared to control. Interestingly, PRL, RDM, and SDM were increased by 66, 58, and 60%, respectively; in NH_4_^+^ fed tobacco plants which were monitored lower compared to NO_3_^-^ supplementation. But, tobacco plants inoculated with T42 fed with NH_4_^+^ nutrient further significantly elevated PRL (84%), RDM (125%), and SDM (72%) compared to control at 70 days, suggesting that T42 participated in nitrogen utilization in NH_4_^+^ fed plant. However, *nia30* mutant showed very limited extent of root growth, root hair density and shoot growth probably due to the fact that double mutant *nia30* line utilized very low nitrate from growth solution (Müller, 1983). Previous studies have shown that nitrate is easily taken up by plant roots improving lateral root growth and architecture (Sun et al., 2015; Ruffel et al., 2016). Here, we demonstrated that LR density was highest in T42 inoculated tobacco plants fed with NO_3_^-^ nutrient. This result is in conformity with the fact that nitrate acts as a signal to trigger number of molecular changes and prevent acidification of root media that help in maintaining membrane potential associated with plant growth (Babourina et al., 2007; Gojon et al., 2011; Giehl et al., 2014). Subsequently, *T. asperellum* T42 inoculation induced modifications in the root architecture in response to N fertilizers that increased total absorptive surface area of roots, and T42 ultimately enhanced R/SDM, especially in NO_3_^-^ supplementation condition.

**Table 1:** Effect of T42 strain on nitrogen nutrient uptake and growth attributes in tobacoo roots.

Plant treatments	Root (total N content) (mg g^-1^ plant^-1^)		RDM (g plant^-1^)		SDM (g plant^-1^)		Primary root length (cm)		Primary root thickness (mm)	
	40 days	70 days	40 days	70 days	40 days	70 days	40 days	70 days	40 days	70 days
Control	32.1 ± 0.8^e^	33.6 ± 0.6^e^	0.9 ± 0.2^c^	1.02 ± 0.1^d^	2.9 ± 0.2^e^	3.8 ± 0.3^e^	16.3 ± 0.9^e^	21.3 ± 1.2^d^	1.0 ± 0.0^b^	1.3 ± 0.3^d^
T42 strain	35.9 ± 0.4^c^	38.1 ± 0.2^c^	1.6 ± 0.1^a^	2.1 ± 0.1^b^c	5.4 ± 0.1^b^c	8.2 ± 0.1^b^	22.5 ± 0.5^b^	34.9 ± 0.7^b^	1.3 ± 0.3^a^b	2.3 ± 0.3^a^b
NO_3_^-^ fed	38.2 ± 0.8^b^	40.3 ± 0.4^b^	1.4 ± 0.1^b^	1.9 ± 0.1^c^d	5.1 ± 0.1^c^	7.4 ± 0.2^c^	20.3 ± 1.2^c^	32.3 ± 1.5^b^	1.3 ± 0.3^a^b	2.0 ± 0.0^a^bc
NO_3_^-^ plus T42 strain	40.7 ± 0.4^a^	44.0 ± 0.5^a^	1.9 ± 0.1^a^b	2.7 ± 0.2^a^	6.4 ± 0.2^a^	9.5 ± 0.2^a^	28.7 ± 0.9^a^	46.0 ± 1.7^a^	1.7 ± 0.0^a^	2.7 ± 0.3^a^
NH_4_^+^ fed	34.1 ± 0.7^d^	35.9 ± 0.2^d^	1.2 ± 0.1^b^	1.6 ± 0.0^d^	4.1 ± 0.1^d^	6.1 ± 0.3^d^	17.0 ± 0.6^d^e	35.3 ± 0.9^b^	1.0 ± 0.3^b^	1.7 ± 0.3^b^c
NH_4_^+^ plus T42 strain	35.8 ± 0.5^c^d	37.4 ± 0.4^c^d	1.7 ± 0.2^a^b	2.3 ± 0.2^b^	5.0 ± 0.1^c^	6.6 ± 0.3^d^	18.7 ± 0.3^c^d	39.3 ± 0.9^b^	1.7 ± 0.3^a^b	2.0 ± 0.0^a^bc
*nia30*	22.4 ± 0.4^g^	23.7 ± 0.3^g^	0.5 ± 0.0^d^	0.7 ± 0.0^f^	0.9 ± 0.1^f^	1.3 ± 0.1^f^	6.7 ± 0.3^g^	10.7 ± 0.3^e^	1.3 ± 0.3^a^b	1.5 ± 0.3^d^
*nia30* plus T42 strain	24.8 ± 0.3^f^	30.8 ± 0.2^f^	0.7 ± 0.0^c^	0.9 ± 0.1^e^	1.2 ± 0.1^f^	1.5 ± 0.1^f^	9.7 ± 0.3^f^	14.3 ± 0.3^d^	1.7 ± 0.3^a^b	2.0 ± 0.0^a^bc

*All values represent mean of eight replicates ± standard error. Total nitrogen content in roots was measured at P < 0.05 level. Different letters denote a statistically significant difference according to Duncan’s multiple range test (at P < 0.05). RDM stands for root dry mass whereas SDM for shoot dry matter.*

**FIGURE 2:**
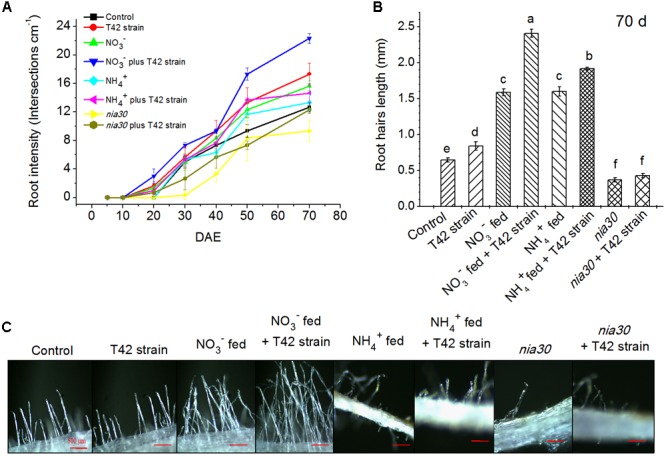
Root hair development in 70 days old tobacco roots. Root hair density (**A**, at *P* < 0.05), root hair length (**B**, at *P* < 0.01) and morphological view (**C**, Bar-500 μm) at 70 days under different conditions. Values are represented as means ± SE (*n* = 3). Different letters on bars indicate significant differences among treatments according to Duncan’s test. DAE stands for days after emergence.

Similarly, root hair development as well as density and length were increased in T42 inoculated tobacco plants compared to control under deprived nutrient condition, after 70 days of plant’s growth (**Figures 2A–C**). But, in presence of the N nutrients, root density and root hairs were more effectively increased in NO_3_^-^ compared to NH_4_^+^ fed. T42 inoculation with tobacco markedly induced both root density and root hairs under NO_3_^-^ and NH_4_^+^ supplementation, especially more in NO_3_^-^. However, T42 inoculation influenced to a limited extent the root hair differentiation, density and length in *nia30* mutant. The results suggested that T42 inoculation with tobacco participated in root proliferation more effectively in NO_3_^-^ nutrient fed condition but did not in the *nia30* mutant.

### T42 Influences Total N Content in Tobacco Roots under Nitrate and Ammonium Nutrition

It has been demonstrated that N is an essential mineral component for crop yields and plant quality (Hajjar and Hodgkin, 2007; Yun et al., 2008; Song et al., 2013; Sun et al., 2015). Although, N inequity in the plant correlates with host-cell sugar export to encourage pathogens susceptibility of French bean (*Phaseolus vulgaris*) to the anthracnose pathogen *Colletotrichum lindemuthianum* (Tavernier et al., 2007). Earlier, it was reported that *Trichoderma* colonizes on the root surface and help in increased nitrogen utilization efficiency and the stored pool of inorganic N plants (Shoresh et al., 2010; Singh et al., 2014; Courty et al., 2015). The putative effect of T42 strain on nitrogen content in tobacco roots under NO_3_^-^ and NH_4_^+^ nutrition was assessed in both wild-type and *nia30* mutant. T42 inoculation with tobacco roots significantly increased total N content by 13% compared to control at 70 days (**Table 1**). N content in tobacco plants fed with NO_3_^-^/NH_4_^+^ nutrient, increased by 20% in NO_3_^-^ and 7% in NH_4_^+^ compared to control. Interestingly, highest total N content was increased by 31 and 11% in T42 inoculated tobacco roots fed in NO_3_^-^ and NH_4_^+^ nutrient, respectively. However, 8% reduction was noticed in *nia30* mutant, compared to control, but T42 inoculated *nia30* plants significantly increased N content from those seen with non- T42 treated (**Table 1**). Thus, NR helped in N acquisition, and T42 treatment effectively promoted this mechanism.

**FIGURE 3:**
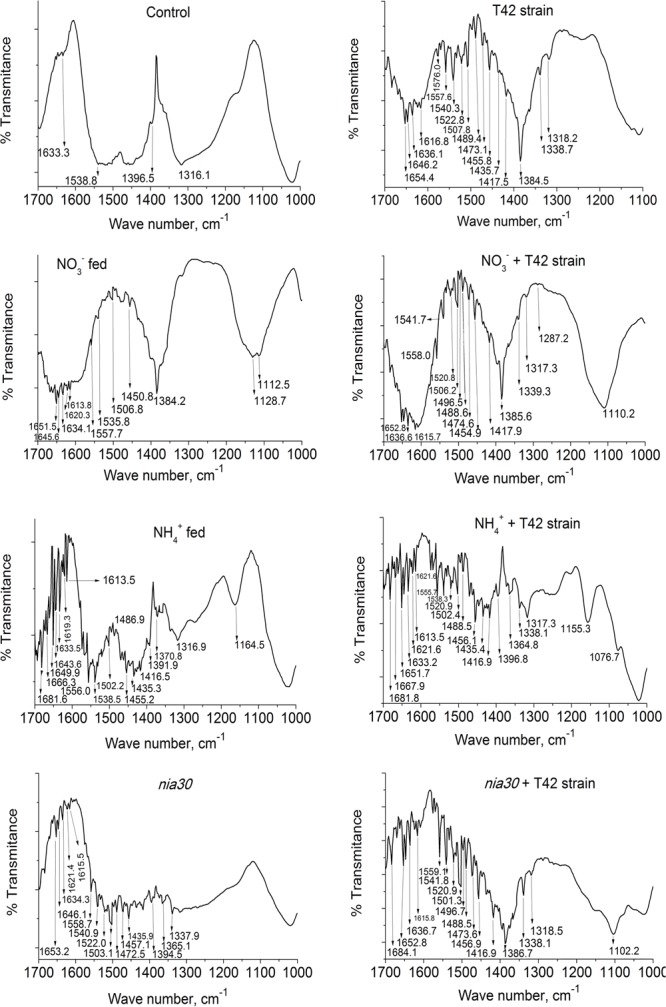
FT-IR spectrum in tobacco roots. Pictorial representation of different signal peaks lies in the range of wave number 1700–1000 cm^-1^ in tobacco root with different treatments: control (used distilled water), T42 strain treated, NO_3_^-^ fed, NO_3_^-^ fed plus T42 bio-primed, NH_4_^+^ fed, NH_4_^+^ fed plus T42 bio-primed, nitrate reductase mutant; *nia30* and *nia30* bio-primed with T42 strain at 70 days after treatment. Values are represented as % transmittance.

Further, FT-IR spectrum was used to observe different functional groups corresponding to each active component present in tobacco root extracts based on the signal peak values (as wave number) appeared in the infrared radiation region. The assignment of bands for their corresponding functional groups are shown in **Figure 3** and Supplementary Table S1. The peaks with highest chance to interfere with nitrate (1410–1340 cm^-1^), NO (1550–1475 cm^-1^) and ammonium (1450–1390 cm^-1^) were observed (Lambert et al., 1987). Comparison of the spectra presented in **Figure 3** shows that there were slight differences in signals between them. The noticeable signal for nitrate was observed after comparing the spectra in 1410–1340 cm^-1^ wave number. The organic compound signal peaks varied due to differences in vibrational tweesting in the band between molecules. Signal peaks in N-O nitro compounds due to symmetric stretching normally lie in the range 1360–1290 cm^-1^ but shifts to 1550–1475 cm^-1^ if bond changes in asymmetric stretching. Similarly, the signal peak for NO_3_^-^ compound lie between 1410 and 1340 cm^-1^ but if it is deformed due to covalent bond formation, then wave number lie in between 860 and 800 cm^-1^. Further, 1450–1475 cm^-1^ wave number is observed for NH_4_^+^ (bending). In the present study, we observed that group frequencies with a strong signal with different treatments appeared in these regions, but the weak signal was monitored in control roots in this range. The results obtained from signal peaks showed the presence of nitrate, NO and ammonium compounds in the root extract (**Figure 3**). Signal peaks in T42 strain pre-treated tobacco roots showed strong functional groups than untreated ones. These results suggested that T42 was able to increase total N content and its assimilation in the form of nitrate, nitro and amino group.

### T42 and N Nutrition Affects NO Production and Release of Cytosolic Ca^2+^ in Tobacco Root

**FIGURE 4:**
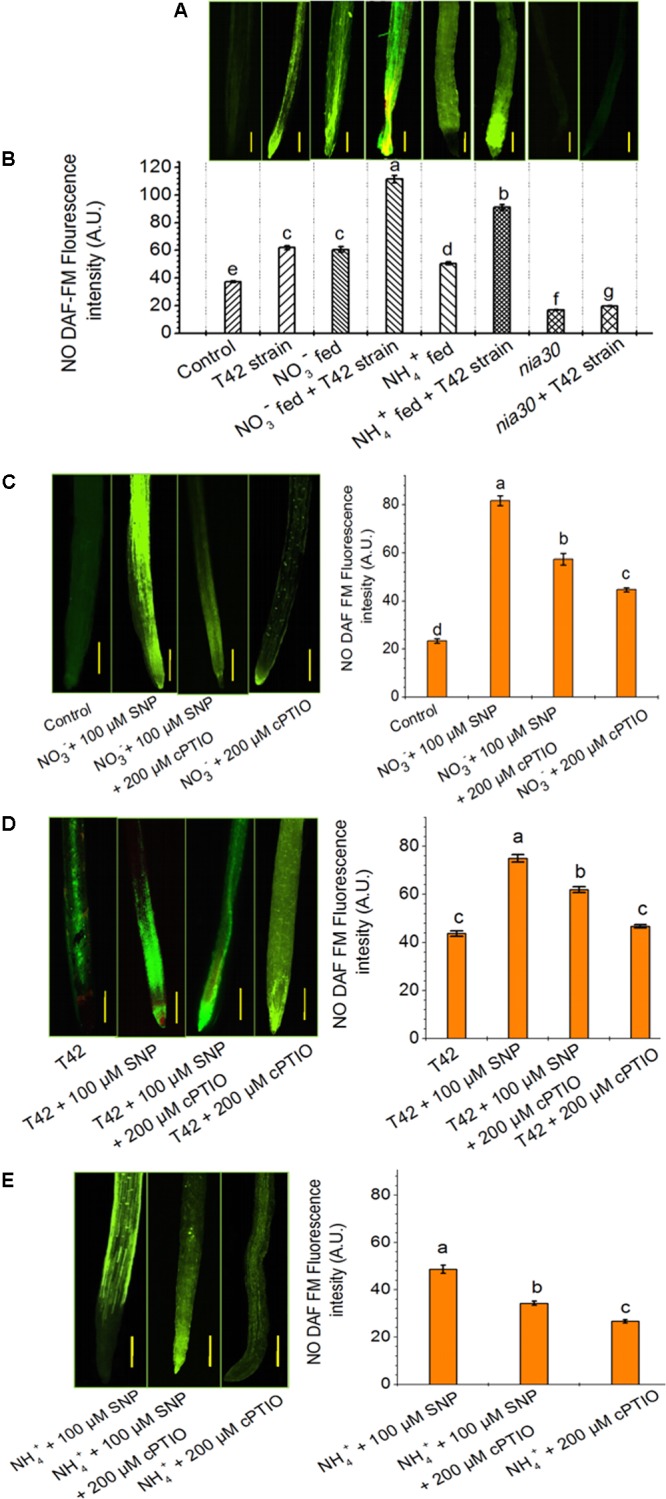
Nitric oxide (NO) production in the root of wild-type and *nia30* mutant tobacco at 70 days. **(A)** NO production, as indicated by green fluorescence detected by DAF-FM staining in roots. The seeds of tobacco bio-primed with T42 were grown under NO_3_^-^ and NH_4_^+^ nutrient supplement, and *nia30* mutant plants (alone) as well as bio-primed with T42 strain were established in presence of nutrient supplements as described in section “Materials and Methods.” **(B)** NO production in root expressed as fluorescence intensity (A.U.) related to the same root regions of **Figure 5B**. NO production and its intensity under the exposure of 100 μM SNP, NO donor, in addition to NO_3_^-^ **(C)**, T42 treatment **(D)** and NH_4_^+^ **(E)** nutrient with control plants for 48 h in 70 days old plants. Application of NO donor in addition to NH_4_^+^ nutrient increased the NO emission in root. The addition of 200 μM cPTIO, NO scavenger reduced fluorescence emission. Values represent the average mean of eleven random selected sites for a definite area from roots (SE; *n* = 11). Letters on bars indicate significant differences in each treatment at *P* < 0.05, as determined by Duncan’s test **(B–E)**.

NO_3_^-^ nutrient is considered as an essential mineral nutrient involved in development in root traits and crop yields. Nitrate also serves as a signal agent which activates several molecular and physiological changes in plant system that lead to increase N pool (Crawford et al., 1995; Stitt, 1999; Gojon et al., 2011). Recently, Trevisan et al. (2011) reported that NO plays a key role in nitrate sensing in maize roots when fed with NO_3_^-^. However, NO production in plants remains controversial whether it is produced through NR or NOS activities. However, application of NO inhibitor and scavenger that interferes with NO biosynthesis provided strong evidence that NO is produced by NR activity (Sun et al., 2015). NR is a key enzyme implicated in NO production under environmental stresses (Freschi et al., 2010; Kolbert et al., 2010). It is known that nitrate is essential for induction of NR expression, but NR dependent NO production in the presence of nitrate nutrient has remained controversial. Moreover, many reports demonstrated that *NIA* expression was essential for NO generation in plants (Planchet et al., 2005; Bright et al., 2006; Sun et al., 2015). However, *nia1/nia2* double mutants for NR deficient showed lower NO level in the root of *Arabidopsis* compared to the wild-type (Schlicht and Kombrink, 2013). This means *NIA* dependent NR is a vital factor for NO production. In this study, the transcript level of *NIA1* dependent *NR* in roots of tobacco increased by seven and threefold under NO_3_^-^ and NH_4_^+^ treatment, respectively, compared to control, which is in agreement with the findings of Yun et al. (2008); however, down-regulation of the same was observed in *nia30* mutant (**Figure 7**). It is possible that NR is essential for increasing NO level in the presence of nitrate nutrient. Application of NO_3_^-^ and NH_4_^+^ supplement enhanced NO accumulation and DAF fluorescence intensity in the tobacco roots, comparatively higher in NO_3_^-^ than NH_4_^+^ (**Figures 4A,B**). Similar results were also demonstrated where consistent accumulation of NO was found in response to nitrate in maize and rice during root development (Manoli et al., 2014; Trevisan et al., 2014; Sun et al., 2015). However, under the nutrient-deprived condition, T42 inoculation increased the expression level of *NIA1* gene, analogous to tobacco roots where another microbe, i.e., *Piriformospora indica* induced NR expression (Sherameti et al., 2005). However, highest transcripts level of *NIA1* was observed in T42 inoculated roots of tobacco fed with NO_3_^-^ nutrient (12-fold) (**Figure 7**). These findings complement with NO generation, where maximum NO production and DAF fluorescence intensity showed in T42 inoculated roots of tobacco fed with NO_3_^-^ nutrient (**Figures 4A,B**). Further, no significant difference in fluorescence intensity was observed in *nia30* mutant, suggesting that NR is essential for NO production and nitrate might be involved in NR-mediated NO production. It was demonstrated that *Trichoderma* spp. inoculation with plants induces NR dependent NO production (Sherameti et al., 2005; Gupta et al., 2014). Thus, we can correlate our findings with those findings and conclude that *T. asperellum* T42 also induced NO production in tobacco roots and the intensity of NO DAF fluorescence was further enhanced in the presence of nitrate nutrient.

**FIGURE 5:**
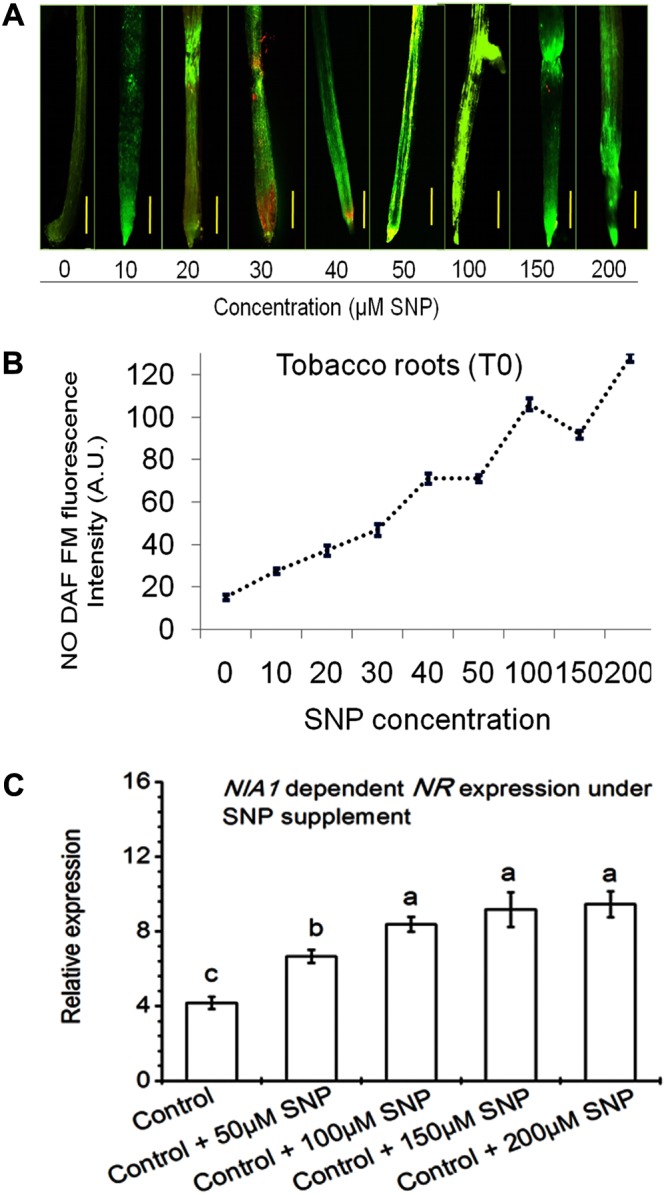
Effects of SNP on *NIA1* dependent NO production in tobacco roots. Different concentrations of SNP (10–200 μM) were applied to 70 days old control tobacco plants under conditions as described in section “Materials and Methods.” DAF- FM Fluorescence was measured by fluorescence microscope **(A)** and intensity **(B)** was expressed as arbitrary units. Transcripts accumulation of *NIA* dependent *NR* gene was analyzed from tobacco after exposure of 50, 100, 150, and 200 μM SNP **(C)**.

In this study, it was shown that NR was involved in NO production in response to nitrate nutrient and T42 in tobacco roots. To further assess the effective role of NO on *NR* expression, 10–200 μM SNP (NO donor) was applied in 70 days old tobacco plants for next 48 h. Highest transcript accumulation of *NIA1* dependent *NR* expression (8.6-fold) and NO DAF fluorescence intensity were at 100 μM SNP (**Figure 5**). It can be concluded that 100 μM SNP was sufficient to induce maximum *NR* expression. A similar observation was also made in cabbage roots where SNP treatment increased the NR activity (Du et al., 2008). These findings also clearly indicated that NO positively stimulates *NR* expression at the post-translational level. However, NO production in NH_4_^+^ fed tobacco roots showed similarity with beech seedlings, where NO production was mediated at post-translational level during NH_4_^+^ uptake (Simon et al., 2009). Moreover, the role of NO in feedback regulation of NO_3_^-^/or NH_4_^+^ nutrient and T42 in root development was further confirmed through application of 100 μM SNP (NO donor) and a marked NO scavenger cPTIO (200 μM) in addition to NO_3_^-^/or NH_4_^+^ nutrient and T42 at 70 days (**Figures 4C–E**). We observed that SNP application elevated extraneous NO accumulation more in NO_3_^-^ nutrient fed tobacco roots compared to T42 inoculation. However, lowest NO DAF fluorescence intensity was noticed in NH_4_^+^ plus SNP condition.

**FIGURE 6:**
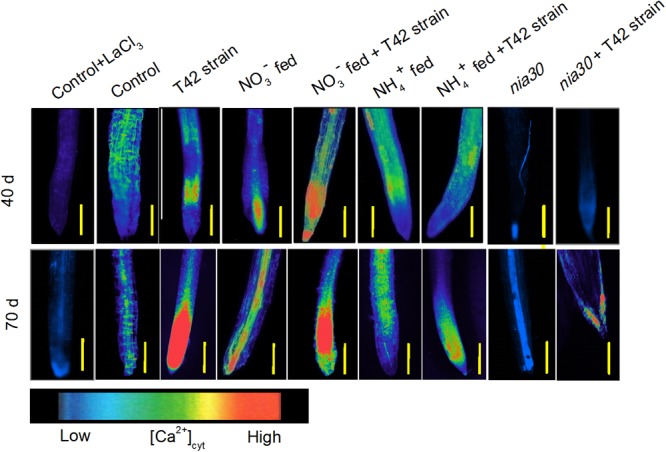
[Ca^2+^]_cyt_ release in the root of wild-type tobacco and *nia30* mutant was stained by 10 μM Fluo-4 AM dye at 40 and 70 days in different treatments. Gradient of color (scale) is used for comparing [Ca^2+^]_cyt_ intensity. Release of cytosolic [Ca^2+^]_cyt_ evaluated with addition of 10 mM Lacl_3_ was used as Ca^2+^ channel blocker with control tobacco roots. Bars are represented as 500 μm.

Several reports have been described in plants where NO-induced [Ca^2+^]_cyt_ second messenger in the cGMP-dependent signaling pathways that help in cell wall synthesis and cellular homeostasis (Lamattina et al., 2003). Presence of Ca^2+^ in plants is associated with root cell and root hair elongation (White, 2001), while its deficiency causes hollow heart in potato (Palta, 2010). In the present study, we checked whether induced NO was linked to Ca^2+^ mediated root formation (Pagnussat et al., 2003). Roots were incubated with inhibitor solution prior to monitoring the stimulus-induced response of plasma membrane Ca^2+^ blocker lanthanum (III) chloride. **Figure 6** shows that lanthanum remarkably reduced the relative fluorescence intensity for [Ca^2+^]_cyt_ compared to control when incubated in the absence of inhibitor. Moreover, results obtained from different treatments in tobacco roots showed that T42 inoculation stimulated a release of relative fluorescence intensity for [Ca^2+^]_cyt_ from 40 to 70 days either from mitochondria or ER to cytosol compared to control. However, the relative fluorescence intensity for [Ca^2+^]_cyt_ was affected only slightly in T42 inoculated *nia30* mutant. NO_3_^-^ nutrient fed tobacco root (alone) was responsible for induced [Ca^2+^]_cyt_ release, similar to tobacco cells where NO influenced Ca^2+^ influx across the plasma membrane (Lamotte et al., 2004). In contrast, NH_4_^+^ supplementation stimulated lesser extent of Ca^2+^ release as compared to NO_3_^-^ nutrient (**Figure 6**). Our findings concluded that NO was generated in response to nitrate nutrient might possibly due to induced [Ca^2+^]_cyt_ production in tobacco roots.

### Activation of Nitrate/Ammonium Transporters Enhanced N Accumulation

**FIGURE 7:**
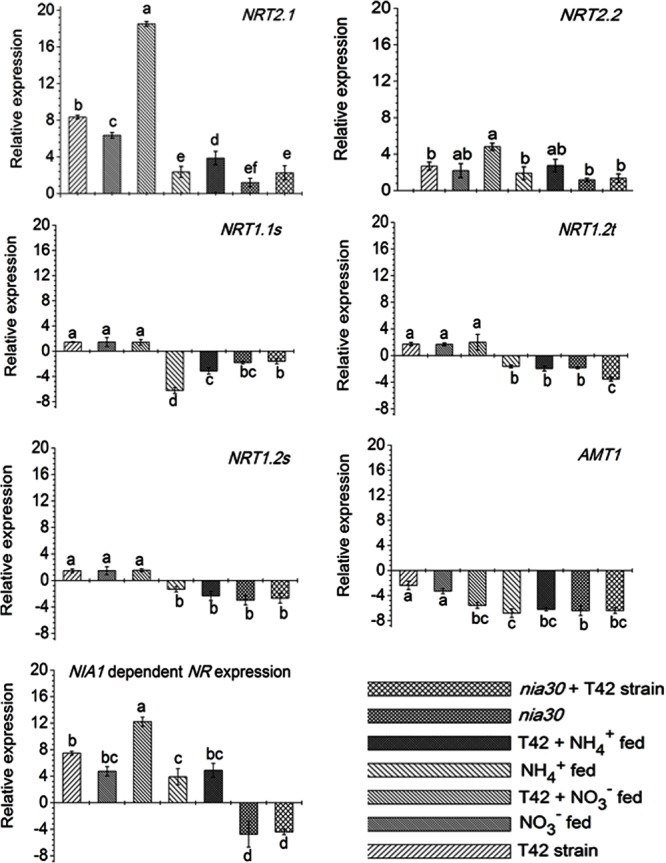
Transcript level of high affinity nitrate transporters (*NRT2.1* and *NRT2.2*), low affinity transporters (*NRT1.1s, NRT1.2s*, and *NRT1.2t*), ammonium transporter (*AMT1*) and *NR* involved in N uptake in roots. Bars represent as SD (Standard Deviation) from means of three biological replicates. Different letters on bars show significant differences as analyzed by Duncan’s multiple rang test (at *P* < 0.01).

**FIGURE 8:**
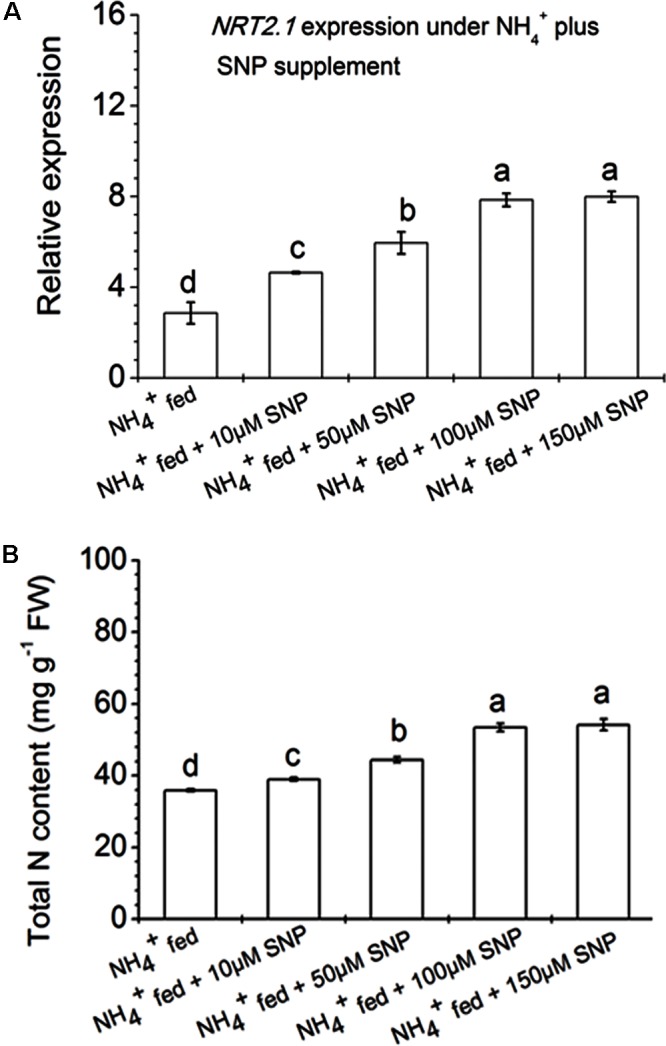
Relative expression of high affinity nitrate transporter *NRT2.1* gene and total nitrogen content in the roots of tobacco. Plants were grown hydroponically under NH_4_^+^ nutrient solution as described in section “Materials and Methods,” and for assessment of effect of high-affinity nitrate transporter (*NRT2.1*) in N uptake, added 10, 50, 100, and 150 μM SNP in NH_4_^+^ nutrient solution for 48 h in 70 days old tobacco plants **(A)**. Total nitrogen content was estimated with same treatments through Kjeldahl method after exposure of 48 h **(B)**. Data represented as the mean ± standard deviation (*n* = 4), bars with different letters represent significant differences as analyzed by Duncan’s multiple range test at *P* < 0.05.

Root architecture development depends on signals derived from nitrogen acquisition and assimilation (Corpas and Barroso, 2015). Stitt (1999) reported that nitrate signaling elevated *NR* expression and NRTs. But high nitrate fertilization preferentially inhibits root: shoot ratio and reduced lateral root formation (Grime et al., 1991). However, limited application of nitrogen fertilization leads to a proliferation of lateral root distribution. Nonetheless, the regulatory mechanism and controlling system of N acquisition as well as assimilation are not fully known in any single plant species. However, several factors that influence at the transcriptional level of the gene involved during nitrate acquisition have been identified in many plant species (Pii et al., 2014, 2016a; Sun et al., 2015; Vidal et al., 2015). In this context, several nitrate and AMTs were classified on the basis of nitrate/ammonium uptake affinity in plant roots (Pii et al., 2014). Few previous reports suggested that *NRT2.1* and *NRT2*.*2* participated in primary root development in maize and rice under nitrate nutrition (Sorgona et al., 2011; Sun et al., 2015; Pii et al., 2016a). In the current investigation, we focused on the expression pattern of different nitrate (viz. *NRT2.1, NRT2.2, NRT1.1s, NRT1.2s, NRT1.2t*) and ammonium (*AMT1*) transporters in roots of tobacco grown under different conditions (**Figure 7**). The transcripts level of two high-affinity NRTs viz., *NRT2.1* and *NRT2.2* were up-regulated during N availability, and particularly more with NO_3_^-^ fed condition compared to NH_4_^+^ fed condition in *Arabidopsis* (Noguero and Lacombe, 2016). T42 treatment under nutrient deprived condition induced transcripts level of most of the genes except *AMT1* in roots at 70 days, but significantly more in case of the *NRT2*.*1* gene (**Figure 7**). Similarly, inoculation of T42 into tobacco roots and fed with N nutrition, up-regulated all those transporters except *AMT1* in NO_3_^-^ nutrition. *NRT2.1* was highly up-regulated, i.e., 18-fold whereas, the same was up-regulated by only 4-fold in NH_4_^+^ fed condition. Besides, induction of *NRT1* and *NRT2* transporters, acts as an inducible high affinity NRTs; another worthy NRT, i.e., *NRT3*, analogous to *NRT2* in plant roots, independently expressed during “induction” phenomenon in response to NO_3_^-^ treatment, which might be involved NO_3_^-^ acquisition in our experiment as well (data not shown) (Okamoto et al., 2006; Pii et al., 2014, 2016a). Transcriptional regulation patterns of these genes gave an overall picture but not a complete picture regarding regulation of the nitrate/AMTs in response to NO_3_^-^/NH_4_^+^ nutrient fed condition in tobacco roots inoculated with *T. asperellum* T42. Likewise, the role of *T. asperellum* T42 in higher expression of NRTs, another filamentous fungi, i.e., *T. harzianum* has been identified in plant roots which enhanced di/tri-peptide transporters (*PTR2*) under nitrogen starvation (Vizcaíno et al., 2006). Further investigating on how each component can show a discrepancy during N availability in the presence of *Trichoderma* spp. may reveal more interesting facts. Moreover, in this work, we observed that the transcript accumulation of *NRT2.1* and *NRT2.2* transporters were greater among all transporters and mutational study with *nia30* tobacco plants further showed that only these two NRTs were responsible for partial nitrate uptake in the *nia30* mutant (Müller, 1983). In addition to NO_3_^-^ and T42 effects, we also examined how NO could elevate expression level of *NRT2.1* and N accumulation in addition to NH_4_^+^ nutrient supplementation (**Figure 8**) through application of 10–150 μM SNP (NO donor) with NH_4_^+^ fed tobacco plants. Interestingly it was observed that expression of high-affinity transporter (*NRT2.1*) and total N content in NH_4_^+^ fed tobacco roots were increased significantly by SNP application. The results thus indicated that increase in N uptake is through NO regulated N acquisition and assimilation.

## Conclusion

The quantitative analysis of different root traits provides novel insights into plant–microbe interactions and its long-term effects on N acquisition and assimilation. Exogenous supplement of N source(s) triggers the plant nutritional status exerting a major impact on individual root traits that have impacts on plant development. On top of that, nitrate supplement can stimulate individual root development and *T. asperellum* T42 helped in nitrogen utilization efficiency. These interesting data raise the question of the genetic control of nitrogen uptake and utilization efficiency on plant growth. The identification of corresponding *Trichoderma* based induction of transcriptional promoters for NRTs will uncover new molecular actors of N-controlled plant growth.

## Author Contributions

Conceived and designed the experiments, performed the experiments, and wrote the paper: PD and BNS. Contributed reagents/materials/analysis tools/equipment facilities: PD, BKS, and HS. Analysis of data: BNS, PD, and GS.

## Conflict of Interest Statement

The authors declare that the research was conducted in the absence of any commercial or financial relationships that could be construed as a potential conflict of interest. The reviewer YP and handling Editor declared their shared affiliation.

## Footnotes

1 http://frodo.wi.mit.edu/primer3/

## Abbreviations

AMT: ammonium transporter
CMC: carboxymethyl cellulose
cPTIO: 2-(4-carboxyphenyl)-4,4,5,5-tetramethylimidazoline-1-oxyl-3-oxide
DAF-FM DA: fluorophore 4, 5-diaminofluorescein-FM diacetate
Fluo-4 AM: 4-(6-Acetoxymethoxy-2,7-difluoro-3-oxo-9-xanthenyl)-4′-methyl-2,2′-(ethylenedioxy)dianiline-N,N,N′, N′-tetraacetic acid tetra -kis (acetoxymethyl) ester
MES-KOH: 2-(N-Morpholino) ethanesulfonic acid sodium salt, 4-Morpholineethanesulfonic acid sodium salt
NRT: nitrate transporter
PPDF: photosynthetic photon flux density
