# Supplementary material for: Trichoderma asperellum T42 Reprograms Tobacco for Enhanced Nitrogen Utilization Efficiency and Plant Growth When Fed with N Nutrients
Source: Front Plant Sci. 2018 Feb 20;9:163. doi: 10.3389/fpls.2018.00163 (PMC5829606; doi:10.3389/fpls.2018.00163)
Supplement: Supplementary file 1 [file Table_1.DOCX]

**Supplementary data**

**TABLE S1:** Assignment of FT-IR bands to functional groups present in *Nicotiana tabaccum* roots extracts. FT-IR data (wave numbers) observed from roots samples match with standard wave number (Source: Lambert et al., 1987)

| Range (Wave number, cm^-1^) | Groups and class of compound | Assignment and remarks | Roots (Wave number, cm^-1^) | | | | | | | |
| --- | --- | --- | --- | --- | --- | --- | --- | --- | --- | --- |
|  |  |  | Control | T42 | NO_3_^-^ | NO_3_^-^  + T42 | NH_4_^+^ | NH_4_^+^ + T42 | *nia30* | *nia30* + T42 |
| 3450-3250 | -OH group in alcohals and phenols | O-H stretch |  |  |  |  |  |  |  |  |
| 3360-3340 | -NH_2_ in primary amines | NH_2_ antisym stretch (solids) |  |  |  |  |  |  |  |  |
| 3000-2850 | -C-H In alkanes | C-H Stretch |  |  |  |  |  |  |  |  |
| 2936-2913 | CH_3_, -CH_2_ -In aliphatic compounds | CH_3_, CH_2_-Anti-symmetric stretch | |  |  |  |  |  |  |  |
| 1724-1700 | C=O In carboxylic acids, ketones | C=O Stretch |  |  |  |  |  |  |  |  |
| 1650-1580 | N-H, 1^O^ Amines | N-H Bend | 1643.7  1633.3 | 1646.2 | 1634.1  1645.6 |  | 1649.9  1643.4 | 1651.7  1573.0 | 1653.2  1646.1 | 1652.8 |
| 1600-1520 | NH_3_^+^ in NH_4_OH | NH_3_ Deformation | 1538.8 |  |  |  |  |  |  |  |
| 1640-1580 | NH_3_^+^ in amino acids | NH_3_ Deformation |  |  | 1620.3 | 1636.6 | 1633.4 | 1633.2 | 1634.3 | 1636.7 |
| 1618-1498 | Benzene ring in aromatic compounds stretch | C=C Aromatic ring | 1506.1 | 1616.8 | 1613.3 | 1615.7 | 1613.5 | 1613.5 | 1615.5 | 1615.8 |
| 1610-1580 | -NH_2_ in amino acids | NH_2_ deformation; broad band |  |  |  | 1608.7 |  |  |  |  |
| 1590-1580 | -NH_2_ primary alkyl amide | NH_2_ deformation (Amide II band) | |  |  |  |  |  |  |  |
| 1575-1545 | -NO_2_ in aliphatic nitro compounds | NO_2_ antisym stretch |  | 1557.6 | 1557.7 | 1558.0 | 1556.0 | 1555.7 | 1558.7 | 1559.1 |
| 1550-1490 | NH_3_^+^ amino acids or hydrochlorides | NH_3_^+^ deformation |  | 1489.4  1540.3 |  | 1496.5 |  | 1538.3 | 1540.9 | 1541.8 |
| 1550-1475 | N-O Nitro compounds | N-O Asymmetric stretch | 1506.1 | 1497.5  1507.7 | 1506.8  1535.8 | 1474.6  1506.2  1541.7 | 1502.3  1519.0  1638.1 | 1502.4  1488.5  1520.9 | 1503.1  1487.9  1522.0 | 1501.3  1520.9  1488.5 |
| 1530-1450 | N=N-O in azoxy compounds | N=N- antisym stretch |  | 1455.8  1473.1 | 1450.8 | 1520.8 |  |  | 1457.1  1472.5 | 1456.1 |
| 1360-1290 | N-O Nitro compounds | N-O Symmetric stretch | 1361.1 | 1338.7 |  |  |  |  |  |  |
| 1420-1400 | C-N in Primary amides | C-N stretch (Two bands) |  | 1417.1 |  | 1517.9 | 1416.3 | 1416.9 | 1417.0 | 1416.9 |
| 1360-1320 | NO_2_ in aromatic nitro compounds | NO sym stretch |  | 1338.7 |  | 1339.3 | 1360.3 | 1338.1 | 1337.9 | 1338.1 |
| 1350-1280 | N=N-O in azoxy compounds | N=N-O sym stretch |  | 1286.7  1318.2 | 1319.4 | 1317.3 | 1316.9 | 1317.3 |  | 1118.5 |
| 1230-1100 | C-C-N in aimnes | C-C-N bending |  |  | 1128.7 |  |  |  |  |  |
| 1300-1150 | Alkyl halides | C-H wag (-CH_2_X) | 1167.8 | 1242.3 | 1235.0 | 1287.2 | 1164.5  1271.1 | 1155.3 |  |  |
| 1120-1030 | C-NH_2_ in primary aliphatic amines | C-N stretch |  |  | 1112.5 | 1110.2 |  |  |  | 1102.2 |

**TABLE S2:**

List of the genes analyzed by means of Real Time qPCR. Tobacco NCBI accession numbers and primer sequences (Nt represents *Nicotiana tabaccum*; NRT- nitrate transporter; AMT- ammonium transporter; *NIA1*- nitrate dependent nitrate reductase)

| Gene names | Accession no. (NCBI) | Primers sequence |
| --- | --- | --- |
| *NtNRT1.2s* | AB102807 | F5'–GCCCTATACATGACGGCGCT-3' |
|  |  | R5'–GCACCGAGCGAGCCTACAT-3' |
| *NtNRT1.2t* | AB102808 | F5'–ATCCCAAGCCTACGACCACCA-3' |
|  |  | R5'–GCACCGAGCGAGCCAACA-3' |
| *NtNRT1.1s* | AB102805 | F5'–TCCCAAGCCTACGACCACCA-3' |
|  |  | R5'–GTCACTGCACCAAGGGATCCC-3' |
| *NtNRT2.1* | AJ557583 | F5'–GGTAATGCCGGAGTTGCTTCCG-3' |
|  |  | R5'–CTCCAGCCGACGACACAAATGAC-3' |
| *NtNRT2.2* | AJ557584 | F5'–CGGTCGATTCTGAACACAAGGCC-3' |
|  |  | R5'–CAACCCCAGCATTACCAACGTCC-3' |
| *NtAMT1* | KJ874416 | F5'–ATCGTCACCTTGTTCGGCCGT-3' |
|  |  | R5'–AGCTGCCCAGAACCCACAGAC-3' |
| *NtNIA1* | X14058.1 | F5'–AACCGCCGTTGAACCGTCTCA-3' |
|  |  | R5'–TCCTTCGGTTGCCAGCACACA-3' |
| *NtL25* | L18908.1 | F5'– GCACCTGGAAGGAACAAACTTGA-3' |
|  |  | R5'- TGCTTTCTTCGTCCCATCAGG-3' |
| *Ntβ-tubulin* | KP316400.1 | F5' –TCAAGGCGGTCAATGCGGTA-3' |
|  |  | R 5'-CTATCCATAGTGCCAGGCTCCAG-3' |
