# Supplementary material for: Trichoderma asperellum T42 Reprograms Tobacco for Enhanced Nitrogen Utilization Efficiency and Plant Growth When Fed with N Nutrients
Source: Front Plant Sci. 2018 Feb 20;9:163. doi: 10.3389/fpls.2018.00163 (PMC5829606; doi:10.3389/fpls.2018.00163)
Supplement: Supplementary file 2 [file Data_Sheet_1.docx]

**SUPPLEMENTARY FIGURE 1:** Screening of forms of N nutrient on the basis of root proliferation

Three weeks old tobacco plantlets were grown hydroponically with different concentration of NO_3_^-^ (10, 50, 100, 150 and 200 mM) and NH_4_^+^ (3, 50, 100, 150 and 200 mM) nutrients as N source for next 30 days. Nutrient media were replaced twice in a week. Root formation (yellow arrows) and root damage (pink arrows) were the priority for screening. On the basis of root proliferation efficiency in different concentrations of NO_3_^-^ and NH_4_^+^ nutrient, number of root initiation and growth were better in 10 mM NO_3_^-^ nutrient supplement. However, root damage was continuously increased with elevated NO_3_^-^ concentration. Maximum root damage was observed in 200 mM NO_3_^-^ nutrient supplement. Similarly, root emergence and growth were observed better in lower concentration of 3 mM NH_4_^+^ nutrient as compared to higher dose.
